# Prognostic role of pre-treatment serum ALB in Patients with oropharyngeal cancer: A retrospective cohort study

**DOI:** 10.3389/fonc.2022.924210

**Published:** 2022-10-13

**Authors:** Jiajia Zhu, Liang Li, Yuansheng Duan, Yansheng Wu, Xudong Wang

**Affiliations:** ^1^ Department of Maxillofacial and Otorhinolaryngology Oncology, Key Laboratory of Cancer Prevention and Therapy, Tianjin Medical University Cancer Institute and Hospital, Tianjin Cancer Institute, National Clinical Research Center of Cancer, Tianjin, China; ^2^ Department of Otolaryngology, Tianjin Children’s Hospital, Tianjin University Children’s Hospital, Tianjin, China

**Keywords:** albumin (ALB), oropharyngeal cancer (OPC), prognosis, overall survival, disease-free survival

## Abstract

**Background:**

The morbidity of oropharyngeal cancer (OPC) is continuing to rise in numerous developed countries. An accurate prognostic assessment is needed to evaluate the malignant degree or risk classification to optimize treatment. Albumin (ALB) as an independent prognostic indicator of cancer survival has been established in previous studies. This study investigated the prognostic value of pre-treatment serum ALB in OPC patients.

**Methods:**

The clinicopathological data of 246 patients diagnosed with OPC from 2010 to 2019 were analyzed retrospectively. Analyze the relationship between ALB and clinicopathological characteristics of patients. The optimal cut-off values for ALB were determined *via* Cutoff Finder (Method for cutoff determination: Survival: significance (log-rank test)). To determine the independent prognostic factors, the Cox proportional hazards model was used to perform univariate and multivariate analyses of the serum ALB levels related to overall survival (OS) and disease-free survival (DFS).

**Results:**

The optimal cut-off point for ALB was 39.15 g/L determined *via* Cutoff Finder. Serum ALB levels were significantly associated with age (P=0.047), Presence of comorbidity (P=0.009), Charlson score index (P=0.007), Hemoglobin (P<0.001), Neutrophil to Lymphocyte Ratio (P=0.002), Albumin-To-Alkaline Phosphatase Ratio (P<0.001), Alkaline phosphatase (P=0.005), T stage (P=0.016), and HPV status (P=0.002). In the univariate and multivariate analyses, ALB was found to be an independent prognostic indicator for DFS (HR =0.39, 95% CI:0.23-0.66, P=0.000) and OS (HR =0.46, 95% CI: 0.25-0.83, P=0.01) in OPC patients.

**Conclusions:**

Pre-treatment serum ALB could serve as a valuable prognostic biomarker for the prognostic stratification of OPC patients.

## Introduction

Oropharyngeal cancer (OPC) has a high rate of morbidity of 11 per 100,000 people and the trend of incidence that has been observed in numerous developed nations continues to rise. Meanwhile, OPC often presents at a more advanced stage at the time of diagnosis due to its ability to grow undetected and its propensity for metastasis (1). The 5-year relative survival rate is only 65%, and Only 29% are diagnosed at an early stage, for which the 5-year survival is 84% (2). For OPC patients, surgery and/or radiation therapy are standard treatments, whereas high-risk or advanced cancers often require synthesized treatment strategies that combinations of surgery followed by radiation or initial concomitant chemotherapy and radiation therapy or target therapy (3, 4). Consequently, an accurate prognostic assessment is needed to evaluate the malignant degree or risk classification to optimize treatment. But in fact, current prognostic models were primarily based on the TNM clinical staging system which remains disputed because of the significant heterogeneity of treatment outcomes in OPC.

In previous studies, a variety of molecular biomarkers is indicated can provide valuable prognostic and predictive information which showed great potential in optimizing the current prognostic stratification system (5, 6). However, some of them are difficult to get and expensive, increasing patients' financial burden and preventing their use in the clinical diagnosis and treatment of OPC.

Albumin (ALB) is the most abundant protein in plasma, accounting for almost half of the total protein content of plasma (7), which is a readily available hematologic index in our clinical practice. ALB also contains a few vital physiologic functions that are required for normal health, including binding, and transporting, maintaining plasm osmotic pressure, and scavenging free radicals, accounts for most of the plasma's antioxidant capacity, impacts the pharmacokinetics of many medications, renders potential poisons harmless (8).

Serum ALB is an independent prognostic factor in various malignancies, like Bladder Cancer (9), pancreatic carcinoma (10), cervical cancer (11), and lung cancer (12). Serum ALB is not a new marker in head and neck cancer. Several papers already mention ALB is related to the survival of HNSCC (13, 14). However, few studies explored the prognostic value between serum ALB and survival in OPC patients.

In conclusion, serum ALB is an easily obtained effective risk-stratification biomarker and is helpful for clinical decision-making regarding human cancers. Therefore, we conducted a retrospective study to examine the relationship between serum ALB levels and survival outcomes, to offer a theoretical foundation for reliable prognostic evaluation.

## Material and methods

### Patients

A total of 246 patients who were histopathologically confirmed oropharynx cancer (OPC) from the years 2010 to 2019 at the Head and Neck Oncology Department of Tianjin Medical University Cancer Institute and Hospital were retrospectively analyzed by us. The Inclusive criteria were patients: (I) with pathologically diagnosed primary OPC; (II) with complete blood cell count before treatment, including the amount of ALB, and (III) with complete clinical and pathological data. The exclusive criteria were patients: (I) who were combined with other systemic malignant tumours; (II) with unreliable clinicopathological data or complete blood cell count before treatment, and (III) with hematologic or immune diseases. Ethical approval was acquired from the Clinical Research Ethics Committee of the Tianjin Medical University Cancer Hospital. A written informed consent agreement was obtained from all patients involved before participating in the study.

### Clinical and pathological data

The data collected in this study were as follows: age, gender, smoking, alcohol consumption, presence of comorbidity, tumour site, tumour size, disease stage (according to the 8th edition of the American Joint Committee on Cancer staging system: The HPV-positive OPC patients were staged according to the HPV-positive staging system; the HPV-negative and HPV-unknown OPC patients were staged according to the HPV-negative staging system), lymph nodes status, HPV status, type of therapy, adjuvant treatments, Charlson’s Comorbidity Index (CCI) and the level of serum Albumin (ALB), Hemoglobin (Hb), Alkaline phosphatase (ALP), Circulating neutrophil cell (CNC), Total lymphocyte cell (TLC) and Total protein(TP). Also, Neutrophil lymphocyte ratio (NLR) was derived from CNC/TLC, Albumin-To-Alkaline Phosphatase Ratio (AAPR) was derived from ALB/ALP and PNI was derived from (ALB g/dl×10) + (0.005×TLC /mm^3^). All patients were pathologically confirmed as squamous cell carcinoma. The laboratory markers’ levels were measured by the Automatic Blood Cell Analyzer (Sysmex, Japan) based on the collected blood samples in the ethylenediaminetetraacetic acid-treated test tubes from each patient who met the inclusion criteria during the week before treatment.

### Treatment

Definitive treatment consisted of surgery and chemo-radiotherapy with curative intent. The primary modalities of surgery or chemo-radiotherapy and postoperative adjuvant therapy were determined *via* consensus of our tumour boards consisting of surgeons, medical oncologists, pathologists and radiologists.

### RT and chemotherapy

The major treatment of patients was based on CCRT (N=127, 51.6%). The specific plan stipulates that all patients are treated with intensity-modulated radiotherapy (IMRT) 1×/day and 5×/week. The prescription dose of primary tumour focuses on the oropharynx is 69.96 Gy/2.12 Gy/33 f. The prescription dose for high-risk lymphatic drainage area is 60.06 Gy/1.82 Gy/33 f. The prescription dose for the low-risk lymphatic drainage area is 50.96 Gy/1.82 Gy/28 f. All patients received cisplatin-based chemotherapy before, during, or after RT.

### Surgical procedure and adjuvant treatment

Surgery included complete extirpation of the primary tumour *via* an open surgical or minimally invasive surgery (including trans-oral endoscopic and trans-oral robotic surgery). There are usually performed *via* a lip-split mandibulotomy or mandibulectomy to arrive at a total tumour removal combined with excision of the surrounding margins. Depending on the tumour location, stage, and clinical nodal metastasis, with or without prophylactic or therapeutic neck dissection. After the resection of OPC, more than 50% of the resections require reconstructions of the defect and involve bony and/or soft tissue reconstruction. Patients with undesirable pathological characteristics underwent surgery and underwent postoperative radiation or chemoradiotherapy **Table 1**.

**Table 1:** Surgical patients’ characteristics.

All Patients (N = 81)		n (%)
General	Gender	
	Male	66 (81.5)
	Female	15 (18.5)
	Age ± SD (years)	57.56 ± 10.72
	ALB± SD (g/L)	41.88 ± 4.34
Cancer details		
	Primary site	
	Tonsil	23 (28.4)
	Base of tongue	37 (45.7)
	Soft palate	21 (25.9)
	Stage	
	I	13 (16.0)
	II	41 (50.6)
	III	24 (29.6)
	IV	3 ( 3.7)
Surgical details		
	Robotic surgery	9 (11.1)
	Open surgery	61 (75.3)
	Endoscopic surgery	11 (13.6)
	Tracheotomy	
	Yes	52 (64.2)
	No	29 (35.8)
	Bilateral/Unilateral Neck dissection	
	Yes	60 (74.1)
	No	21 (25.9)
	Flap reconstruction	
	Yes	45 (55.6)
	No	36 (44.4)
	Mandibulotomy	
	Yes	33 (40.7)
	No	48 (59.3)
Postoperative details		
	Adjuvant RT/CCRT	
	Yes	46 (56.8)
	No	35 (43.2)
	Long-term Gastric-Tube Dependence	
	Yes	55 (67.9)
	No	26 (32.1)

### Follow-up

After the end of treatment, all patients were follow-up for at least 3 years, or until death or disease progression or the study cut-off date (October 2021). The endpoints of the study were determined according to long-term oncological outcomes in the form of disease-free survival (DFS)and overall survival (OS)at a mean follow-up of 40 months (1–129months). Death due to any cause is defined as OS, and the local progression, distant metastasis, or death due to disease progression is defined as DFS.

### Statistical methods

Statistical analyses were performed with R Studio version 4.2.1 (R Foundation for Statistical Computing, Vienna, Austria). We used Pearson’s chi-square test, Yates’ correction test and Fisher’s exact test to compare categorical variables and the Mann-Whitney U test to compare continuous variables [mean ± standard deviation (SD); median and interquartile range (IQR)], respectively. The association between clinicopathologic variables with survival data was determined by the univariate and multivariate cox regression analyses. Univariate and multivariate cox regression analyses were used to determine the independent prognostic factors of OS and DFS. P<0.05 was considered statistically significant. Kaplan-Meier curve with Log-rank test was applied to analyse the differences in OS and PFS among low/high ALB groups.

### Grouping Criteria of ALB

We used the Cutoff Finder (http://molpath.charite.de/cutoff), to determine the optimal cut-off point for ALB in predicting OPC survival. We could then compare clinicopathological characteristics and other nutritional and haematological markers of the two groups of patients stratified by this threshold of ALB levels.

## Results

### Laboratory markers

The mean values regarding ALB, Hb, CNC, TLC, TP and ALP levels in all OPC patients were 42.3 ± 4.05 (g/L), 143.45 ± 15.85 (g/L), 4.26 ± 1.64 (10 ^9^ /L), 1.79±0.66 (10 ^9^ /L), 72.12±6.89 (g/L) and 84.75 ± 24.07 (U/L). In addition, both AAPR, NLR and PNI could thus be obtained with mean values of 0.54 ± 0.17, 2.73 ± 1.58 and 51.26±5.76 respectively, as shown in **Table 2**.

**Table 2:** Baseline characteristics of OPC patients.

Characteristics		ALB		
	Total, n (%)	ALB< 39.15 (N%)	ALB≥39.15 (N%)	P-value
Total	246	48	198	
Albumin (g/L)				
Mean ± SD	42.3±4.05	36.45±2.82	43.74±2.85	<0.001*
Median (IQR)	42.6 (40.0-45.2)	36.95 (36.20- 38.30)	43.40 (41.52- 45.75)	
Age (Years)				0.047*
≤65	197 (80.1)	33 (68.8)	164 (82.8)	
>65	49 (19.9)	15 (31.2)	34 (17.2)	
Gender				0.746
Male	209 (85.0)	42 (87.5)	167 (84.3)	
Female	37 (15.0)	6 (12.5)	31 (15.7)	
Smoking				0.818
Never	71 (28.9)	15 (31.2)	56 (28.3)	
Current/former	175 (71.1)	33 (68.8)	142 (71.7)	
Alcohol consumption				0.997
Never	105 (42.7)	21 (43.8)	84 (42.4)	
Current/former	141 (57.3)	27 (56.2)	114 (57.6)	
Presence of comorbidity				0.009*
Absent	141 (57.3)	19 (39.6)	122 (61.6)	
Present	105 (42.7)	29 (60.4)	76 (38.4)	
Charlson score index				0.007*
0	175 (71.1)	26 (54.2)	149 (75.3)	
>1	71 (28.9)	22 (45.8)	49 (24.7)	
PNI				<0.001*
Mean ± SD	51.26±5.76	43.87±4.22	53.05±4.53	
Median (IQR)	51.75 (47.62-55.05)	44.62 (44.62-46.34)	52.58 (50.11-55.60)	
TP (g/L)				<0.001*
Mean ± SD	72.12±6.89	67.72±7.60	73.19±6.28	
Median (IQR)	72.8 (68.90-75.78)	67.45 (61.98-71.55)	73.65 (70.22-76.08)	
TLC (10^3^/mm^3^)				<0.001*
Mean ± SD	1.79±0.66	1.48±0.50	1.86±0.68	
Median (IQR)	1.77 (1.36-2.14)	1.57 (1.19-1.77)	1.86 (1.41-2.18)	
Hb (g /L)				<0.001*
Mean ± SD	143.45±15.85	132.94±16.76	145.99±14.56	
Median (IQR)	143.5 (134.0- 154.0)	137.0 (123.0- 145.2)	145.0 (136.0- 155.8)	
NLR				0.002*
Mean ± SD	2.73±1.58	3.36±1.81	2.58±1.48	
Median (IQR)	2.22 (1.70- 3.23)	3.080 (1.968- 4.242)	2.17 (1.64- 2.87)	
CNC (10 ^9^ /L)				0.415
Mean ± SD	4.26±1.64	4.43±1.81	4.21±1.59	
Median (IQR)	3.97 (3.14- 5.19)	3.98 (3.19- 5.81)	3.97 (3.14- 4.81)	
AAPR				<0.001*
Mean ± SD	0.54±0.17	0.43±0.14	0.57±0.17	
Median (IQR)	0.52 (0.43- 0.64)	0.41 (0.33- 0.50)	0.5463 (0.46- 0.66)	
ALP (U/L)				0.005*
Mean ± SD	84.75±24.07	93.44±29.30	82.65±22.20	
Median (IQR)	82.0 (68.0- 98.0)	89.0 (72.25- 110.25)	80.0 (68.0- 95.0)	
Site (n, %)				0.096
Tonsil	107 (43.5)	19 (39.6)	88 (44.4)	
Base of tongue	85 (34.6)	13 (27.1)	72 (36.4)	
Soft palate	54 (22.0)	16 (33.3)	38 (19.2)	
Disease stage				0.151
I/II	52 (21.1)	6 (12.5)	46 (23.2)	
III/IV	194 (78.9)	42 (87.5)	152 (76.8)	
T stage				0.016*
T1-2	162 (65.9)	24 (50.0)	138 (69.7)	
T3-4	84 (34.1)	24 (50.0)	60 (30.3)	
Lymph nodes status				0.246
Negative(cN1-3)	76 (30.9)	11 (22.9)	65 (32.8)	
Positive(cN0)	170 (69.1)	37 (77.1)	133 (67.2)	
HPV status				0.002*
Positive	27 (11.0)	5 (10.4)	30 (15.2)	
Negative	48 (19.5)	18 (37.5)	22 (11.1)	
Unknown	171 (69.5)	25 (52.1)	146 (73.7)	
Type of therapy				0.812
Surgery	81 (32.9)	17 (35.4)	64 (32.3)	
CCRT	165 (67.1)	31 (18.8)	134 (81.2)	

PNI, prognostic nutritional index; TP, total protein; TLC, total lymphocyte cell; Hb, hemoglobin; NLR, neutrophil to lymphocyte ratio; CNC, circulating neutrophils cell; AAPR, albumin-to-alkaline phosphatase ratio; ALP, alkaline phosphatase; ALB, albumin; CCRT, concurrent chemoradiotherapy; RT, radiation therapy

*Indicates P < 0.05

### Patient characteristics

**Figure 1:**
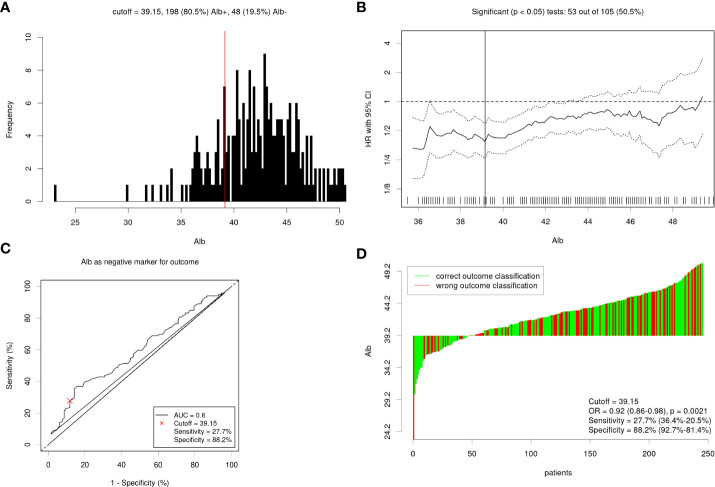
**(A)** Distribution based cutoff optimization (independent of outcome and survival data) in the OPC data. **(B)** Cutoff optimization by correlation with a binary variable (ALB<39.15 g/L; ALB≥39.15 g/L) or survival in the OPC data. **(C)** Receiver operating characteristic curve for albumin levels predicting overall survival. **(D)** Detailed analysis of the optimal dichotomization of the OPC cancer data.

A total of 246 patients with OPC were included in this study. The majority (85%) of patients were male, and 80% were under the age of 65 years. Of the patients, 71.1% had a smoking history and 57.3% had a history of alcohol consumption. The primary tumour sites included the tonsil (43.5%), the soft palate (22.0%), and the base of the tongue (34.6%). Most patients (78.9%) were at an advanced stage (stage III /IV). Concurrent surgery had been received by 32.9% of the patients, and the rest had received radiochemotherapy. Of the patients, 11.0% were HPV positive and 19.5% were HPV negative, however, the rest 69.5% were HPV status unknown. The mean ALB was (42.32±4.05) g /L. The optimal cut-off point for ALB was calculated by Cutoff Finder for OS (**Figure 1**). Then, ALB was transformed into a dichotomous variable according to this cut-off value (<39.15 g/L, n=48; ≥39.15 g/L, n=198). **Table 2** shows the patients’ baseline clinical characteristics according to ALB. A low serum ALB level was related to age (P=0.047), Presence of comorbidity (P=0.0095), Charlson score index (P=0.007), PNI (<0.001), TP (<0.001) , TLC (<0.001), Hb (P<0.001), NLR (P=0.002), AAPR (P<0.001), ALP (P=0.005), T stage (P=0.016), and HPV status (P=0.0021).

### ALB evaluation

The optimal cut-off values for ALB were determined *via* the Cutoff Finder [Method for cutoff determination: Survival: significance (log-rank test)], various cut-off points for ALB were statistically significant for prognostic prediction, and an ALB of 39.15 (Normal range from 35-55 g/L) was finally recognized as the optimal cut-off point, as shown in **Figure 1**. And then the OPC patients were categorized into two groups according to the optimal cut-off value of ALB, respectively.

### Survival analysis

**Figure 2:**
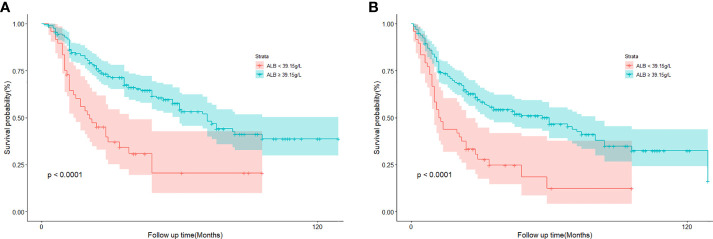
**(A)** Kaplan-Meier curve of cumulative overall survival in 246 patients with oropharyngeal cancer according to albumin levels classification. **(B)** Kaplan-Meier curve of cumulative disease-free survival in 246 patients with oropharyngeal cancer according to albumin levels classification.

We try to explore whether serum ALB level could be an independent prognostic factor for OPC patients. We further investigated associations between different clinical variates with patients’ survival data. The median follow-up time was 34 months (range: 1-129months). A total of 119 (48.4%) patients died and 140 (56.9%) progressed. Kaplan-Meier curve analysis revealed that the OS of patients with low ALB levels (<39.15 g/L) was significantly shorter than that of patients with high ALB levels (≥39.15 g/L) (log-rank P<0.0001) (**Figure 2A**). Also, patients with low ALB levels had significantly shorter DFS than patients with high ALB levels (log-rank P<0.0001) (**Figure 2B**). Univariate and multivariate cox regression analyses were used to ascertain independent prognostic factors of DFS and OS (**Table 3**). Univariate analyses illustrated that Charlson score index (CCI); ALB levels; disease stage; T stage; Lymph nodes status; PNI, NLR and CNC before treatment were significantly associated with poor OS (all P <0.05). In addition, CCI; ALB levels; Alcohol consumption; PNI and CNC before treatment; tumour site; disease stage; T stage. Survival analysis revealed that only ALB was an independent influencing biomarker both of DFS (HR =0.45, 95% CI:0.31-0.68, P=0.000) and OS (HR =0.44, 95% CI: 0.29-0.68, P=0.000) in OPC patients (**Table 3**, **Figures 2A, B**). In addition, Charlson score index (P=0.01), T stage (P =0.02) and lymph nodes status (P=0.04) were independently prognostic parameters for patients’ OS; Site (P=0.01) and CNC (P=0.046) were independently prognostic parameters for patients’ DFS.

**Table 3:** Univariate and multivariate survival analyses between clinicopathologic characteristics and survival on OS and DFS in patients with OPC.

Characteristics	Overall Survival				Progression Free Survival			
	Univariable Analysis		Multivariable Analysis		Univariable Analysis		Multivariable Analysis	
	HR with 95%CI	P Value	HR with 95%CI	P Value	HR with 95%CI	P Value	HR with 95%CI	P Value
Age (Years)								
≤65	Reference				Reference			
>65	1.3 (0.83-1.9)	0.26			1.2 (0.78-1.7)	0.47		
Gender								
Male	Reference				Reference			
Female	0.65 (0.36-1.2)	0.16			0.63 (0.36-1.1)	0.1		
Smoking								
Never	Reference				Reference			
Current/former	1.1 (0.75-1.7)	0.56			1.2 (0.79-1.7)	0.45		
Alcohol consumption								
Never	Reference				Reference			
Current/former	1.4 (0.98-2.1)	0.062			1.4 (1-2)	0.047*	1.5 (1-2.1)	0.036*
Presence of comorbidity								
Absent	Reference				Reference			
Present	1.4 (0.99-2)	0.057			1.2 (0.85-1.7)	0.31		
Charlson score index								
0	Reference				Reference			
>1	1.8 (1.2-2.6)	0.003*	1.7 (1.1-2.4)	0.01*	1.4 (1-2)	0.051*	1.4 (0.96-2)	0.082
ALB (g/L)								
<39.15	Reference				Reference			
≥39.15	0.39 (0.26-0.58)	<0.001*	0.46 (0.25-0.83)	0.01*	0.43 (0.29-0.62)	<0.001*	0.39 (0.23-0.66)	0.000*
PNI (Per Unit Increase)	0.95 (0.92-0.99)	0.00*	1 (0.95-1.1)	0.97	0.97 (0.94-1)	0.04*	1 (0.98-1.1)	0.35
TP (Per 1 g/L Increase)	1 (0.98-1)	0.4			1 (0.99-1)	0.29		
TLC (Per 1 10^9^/L Increase)	0.88 (0.66-1.2)	0.39			1 (0.78-1.3)	0.96		
Hb (Per 1 g/L Increase)	0.99 (0.98-1)	0.16			0.99 (0.98-1)	0.37		
NLR (Per Unit Increase)	1.1 (1-1.2)	0.016*	0.98 (0.82-1.2)	0.84	1.1 (0.99-1.2)	0.066		
CNC (Per 1 10^9^/L Increase)	1.2 (1-1.3)	0.008*	1.1 (0.93-1.3)	0.29	1.1 (1-1.3)	0.012*	1.1 (1-1.2)	0.046*
ALP (Per 1U/L Increase)	1 (0.99-1)	0.55			1 (1-1)	0.44		
AAPR (Per Unit Increase)	0.54 (0.17-1.7)	0.29			0.55 (0.19-1.6)	0.27		
Site (n, %)								
Tonsil	Reference				Reference			
Base of tongue	1.2 (0.94-1.4)	0.18			1.2 (1-1.5)	0.029*	1.3 (1.1-1.6)	0.01*
Soft palate	Reference				Reference			
Disease stage								
I/II	Reference				Reference			
III/IV	2.6 (1.5-4.5)	0.001*	1.2 (0.58-2.6)	0.59	2.4 (1.5-3.9)	0.000*	1.7 (0.85-3.5)	0.13
T stage								
T1-2	Reference				Reference			
T3-4	1.8 (1.3-2.6)	0.001*	1.7 (1.1-2.6)	0.02*	1.6 (1.1-2.2)	0.011*	1.1 (0.7-1.6)	0.76
Lymph nodes status								
Negative	Reference				Reference			
Positive	2.1 (1.4-3.3)	0.001*	1.8 (1-3.3)	0.04*	(1.1-2.4)	0.011*	1.2 (0.67-2)	0.57
Type of therapy								
Surgery	Reference				Reference			
CCRT	1.3 (0.87-1.9)	0.21			1.2 (0.84-1.7)	0.3		
Gastric-Tube Dependence								
Absent	Reference				Reference			
Present	0.98 (0.64-1.5)	0.91			1.1 (0.71-1.6)	0.77		
Neck dissection								
Absent	Reference				Reference			
Present	0.92 (0.61-1.4)	0.72			0.97 (0.66-1.4)			
Postoperative RT/CCRT								
Absent	Reference				Reference			
Present	0.96 (0.61-1.5)	0.87			1.3 (0.84-1.9)	0.27		
RT								
Absent	Reference				Reference			
Present	0.74 (0.51-1.1)	0.11			0.89 (0.64-1.3)	0.51		
CCRT								
Absent	Reference				Reference			
Present	0.77 (0.53-1.1)	0.16			0.89 (0.63-1.2)	0.49		

*Indicates P < 0.05.

## Discussion

In this study, we aimed to investigate the correlation between pre-treatment serum ALB and the prognosis of OPC. We found that lower pre-treatment serum ALB levels were independently associated with a worse prognosis for OPC patients. These results verified that pre-treatment serum ALB might serve as a valuable prognostic biomarker for the prognostic stratification of OPC patients.

As we all know, nutritional status influences disease course, prognosis and postoperative recovery of head and neck cancer (HNC) (15, 16), with the consequences of delayed wound healing, long-term gastric tube dependence, increased complications, and postoperative infections. Serum ALB is one of the most popular ways to evaluate cancer patients' nutritional status among the several available approaches (17). Several studies have reported that a lower serum ALB value was linked to a shorter DFS and OS in a variety of cancers, however, only a few studies have focused on the clinical impact on the level of serum albumin in head and neck cancer patients (13, 18–20). Consequently, there are limited data exploring the prognostic value between serum ALB levels and survival data of OPC, and the biological mechanisms of actions are yet unclear.

In previous research, 30–60% of head and neck cancer patients have malnutrition (21), caused by complex factors, such as odynophagia, dysphagia, mechanical obstruction, abnormal motility of the deglutition muscles and anorexia (22). The oropharynx is an important part of the aerodigestive tract, in this study most of the tumours were located in the tonsil (43.5%), the base of the tongue (34.6%), and most OPC patients were in an advanced stage (78.9%). All patients in our current research are not cachectic, and before therapy, the median values of the majority of baseline blood nutrition indices like PNI, TP, Hb, and ALB were within the normal range. But some patients may exist lower ALB levels or be mild malnourished before treatment due to the food-intake disorder, increased tumour-induced protein consumption and a loss of appetite. However, as a result of oral intake limits increased after treatment and enhanced protein catabolism brought on by aggressive surgery, low levels of ALB and malnutrition may further develop. Thus, without careful consideration in malnourished and low ALB cases before surgery may lead to serious complications and an increased risk of long-term gastric tube dependence after surgery, to improve the prognosis of OPC surgery, an assessment of nutrition conducted before and after surgery is vital.

On the other hand, the acute toxicity of chemotherapy and radiation was significant (23), and the radio-sensitizing impact of chemotherapy may also lead to higher acute toxicity and late complications (24). In addition to mucositis, an increased radiation dose may lead to hyperactivation of transforming growth factor β1 (TGFβ1) bringing about excessive fibrosis (25), which may be responsible for impaired oral feeding and prolonged dependence on tube feeding. Thus, the patient’s life quality and food intake may be adversely affected (26). The majority of patients (67.1%) in our study performed chemo-radiotherapy therapy from the beginning. Oral mucositis and dysphagia occurred frequently, which may prevent the patient from oral feeding and result in malnourishment and low serum albumin levels.

ALB plays a key role in the antioxidant capacity in human plasmas, which can render potential toxins harmless and remove reactive oxygen species (ROS) (7, 27), which is a vital antioxidant in the body. Various cell signalling pathways that have been connected to cellular inflammation, transformation, tumour proliferation, angiogenesis, invasion and cancer metastasis are modulated by ROS (25, 28), which play a crucial role in tumorigenesis (29). When an imbalance in the production of ROS and the antioxidant defences, there will be oxidative stress. And then, oxidative stress will promote damage to the cell structure including proteins, lipids, membranes and DNA (30), it promotes tumour progression and further decreases levels of antioxidants such as serum ALB. Simultaneously, radiotherapy and chemotherapy both result in oxidative stress, although in distinct ways. Chemotherapeutic drugs promote cytotoxicity by upregulating ROS *in vitro* or by increasing mtROS *via* the creation of mitochondrial DNA adduct (31, 32); Radiation therapy causes free radicals to damage DNA and depletes GSH, which in turn causes an increase in ROS (33, 34). Therefore, those patients who received CCRT treatment may lead to a rise in oxidative stress and a drop in antioxidants while the destruction of tumour cells (35, 36). In our study, most patients had chemo-radiotherapy treatment from the start. Although, ROS could serve as adjuncts to improve the toxicity of chemotherapy and enhance radiosensitivity to reduce the development of resistance (35, 37). However, the survival benefit of those patients undergoing chemo-radiotherapy may be accompanied by a decrease in serum ALB levels and enhanced oxidative stress. In addition, oxidative stress and inflammation are two interrelated pathophysiological processes that are intimately associated (38). At the location of the inflammation, inflammatory cells release several reactive species, which intensify oxidative stress (39). On the other hand, several reactive oxygens species can start a cascade of intracellular signals that promote the production of pro-inflammatory genes (40). Indeed, lower serum ALB levels are closely related to systemic inflammation response in cancer patients (41, 42). The hepatic acute-phase protein response is one of the key metabolic alterations induced by proinflammatory cytokines, CRP and ALB were principle positive and negative acute-phase proteins, respectively (43). Proinflammatory cytokine, tumour necrosis factor (TNF), interleukine-1(IL-1) and interleukine-6 (IL-6) have been demonstrated to influence ALB levels in the bloodstream as a result of inflammation (44), which increases the demand for amino acids *via* stimulating the synthesis of CRP in the liver and inhibitors liver production of ALB (45, 46). The activation of systemic inflammation by malignancies will cause anorexia and increased catabolic loss of protein and eventually contribute to cancer-related malnutrition, which is a major cause of such patient deterioration (47). Conversely, serum ALB levels decreased could damage the human immune system (48). And then, the weakened immune system may increase the risk of infection and trigger a systemic inflammatory response. Infections hurt nutritional status and malnutrition will impair infection resistance. Systemic inflammation and malnutrition are exacerbated, and the vicious circle repeats itself.

Serum ALB levels are not only a window into the patient’s nutritional status but also a useful factor for predicting the prognosis of a patient with cancer which can be readily measured in resource-limited settings and will be critical for future clinical intervention studies in cancer patients.

## Limitation

The primary limitation of this study was that it was retrospective research of a single-centre cohort with small sample size. Exclusive a lot of data incomplete cases inevitably led to selection bias. Data on p16 and HPV status were only obtained for some

patients, the patients with HPV-positive OPC are quite different from the regular OPC population, further studies need the analyses nutritional and hematologic markers according to the HPV status. To confirm our findings, prospective studies with a strict research design in terms of pre-treatment serum ALB and substantial sample size are needed in the future.

## Conclusion

The current study investigated the prognostic value of serum ALB for OPC patients. We showed that low pre-treatment ALB levels in the serum were related to poor survival, and that low ALB levels were an independent predictor of poor DFS and OS. As a result, the measure of the pre-treatment serum ALB level might be utilized in clinical trials to better identify cancer patients' baseline risk. The combination of serum ALB levels and the TNM clinical staging system can offer a theoretical foundation for the future advancement of prognostic classification of OPC patients to guide individualized treatment or necessary nutritional interventions.

## Data availability statement

The original contributions presented in the study are included in the article/supplementary material. Further inquiries can be directed to the corresponding authors.

## Ethics statement

The studies involving human participants were reviewed and approved by the ethics committee of the Tianjin Medical University Cancer Institute and Hospital (No. bc2020110). The patients/participants provided their written informed consent to participate in this study. Written informed consent was obtained from the individual(s) for the publication of any potentially identifiable images or data included in this article.

## Author contributions

JZ: conception of the work, data collection, data analysis and interpretation, drafting the article. LL: data collection, data analysis and interpretation. YD: critical revision of the article. XW and YW: conception of the work and final approval of the version to be published. All authors contributed to the article and approved the submitted version.

## Funding

The Science&Technology Development Fund of Tianjin Education Commission for Higher Education (grant no. 2016YD15).

## Acknowledgments

We give special thanks to two reviewers for their positive comments and constructive suggestions for this manuscript.

## Conflict of interest

The authors declare that the research was conducted in the absence of any commercial or financial relationships that could be construed as a potential conflict of interest.

## Publisher’s note

All claims expressed in this article are solely those of the authors and do not necessarily represent those of their affiliated organizations, or those of the publisher, the editors and the reviewers. Any product that may be evaluated in this article, or claim that may be made by its manufacturer, is not guaranteed or endorsed by the publisher.
